# The role of classical protein tyrosine phosphatases in diabetic kidney disease and therapeutic implications

**DOI:** 10.1186/s12929-026-01282-7

**Published:** 2026-09-02

**Authors:** Grace LeBleu, Fawaz G. Haj

**Affiliations:** 1 Department of Nutrition, University of California Davis, Davis, CA USA; 2 Comprehensive Cancer Center, University of California Davis, Sacramento, CA USA; 3 Division of Endocrinology, Diabetes, and Metabolism, Department of Internal Medicine, University of California Davis, Sacramento, CA USA

**Keywords:** Protein tyrosine phosphatase, Tyrosine phosphorylation, Diabetic nephropathy, Diabetic kidney disease, Pharmacological inhibitors, Therapeutic interventions

## Abstract

Approximately one-third of people with diabetes have diabetic kidney disease (DKD), and DKD remains a leading cause of kidney failure in the United States. DKD is characterized by injury to the glomerular filtration barrier and renal tubulointerstitium, arising from complex and convergent metabolic, hemodynamic, and inflammatory insults. Tyrosine phosphorylation, a reversible post-translational modification, is a central regulatory mechanism in these pathways and is dynamically controlled by protein tyrosine kinases (PTKs) and protein tyrosine phosphatases (PTPs). Aberrant tyrosine phosphorylation is a hallmark of DKD and implicates PTPs as key modulators of podocyte, endothelial, tubular, and immune cell dysfunction. Over 100 genes code for PTPs in humans, with 38 ‘classical’ PTPs that specifically target tyrosine motifs, including receptor-type and non-receptor-type subtypes. Several classical PTPs are implicated in DKD pathogenesis, while others are increasingly associated with signaling cascades that drive renal metabolism, inflammation, fibrosis, and cell death. There is growing interest in PTPs as their contributions to the disease emerge, though the role(s) of some remain uncharacterized in DKD. Moreover, recent breakthroughs in pharmaceuticals targeting PTPs for metabolic diseases and cancer have ignited interest in repurposing or tailoring PTP‑directed therapeutics for DKD. This review summarizes current knowledge and recent advances of classical PTPs in DKD, including their roles in insulin signaling, cellular architecture, and glomerular and tubular cell injury. It further discusses existing DKD treatments and highlights emerging strategies to pharmacologically modulate PTP activity, such as active‑site inhibitors, allosteric agents, and antibody‑targeting approaches. By integrating mechanistic insights with drug discovery efforts, this review outlines how targeting specific PTPs could complement current standard‑of‑care therapies and open new avenues for precision treatment of DKD.

## Background

Diabetic kidney disease (DKD) is a major microvascular complication of diabetes, affecting 20–40% of individuals with diabetes. It is driven by chronic metabolic and hemodynamic stressors, including hyperglycemia, and progresses through a complex, multifactorial pathophysiological process that remains incompletely understood. Diagnostic criteria for DKD include albuminuria and/or a reduced estimated glomerular filtration rate (eGFR) in the absence of other identifiable causes of kidney disease [1]. The clinical course of DKD has classically been described as progression from asymptomatic glomerular hyperfiltration to the onset of albuminuria, followed by overt proteinuria and progressive decline in glomerular filtration rate (GFR), and ultimately culminating in end-stage renal disease that necessitates renal replacement therapy, such as dialysis or transplantation. DKD remains a leading cause of kidney failure worldwide, with its global burden expanding dramatically in recent decades. Between 1990 and 2021, the global burden of chronic kidney disease due to type 2 diabetes (T2DM) nearly doubled, reflecting an approximate 80–90% increase [2]. As kidney function declines, healthcare utilization and expenditures rise sharply, reflecting the substantial economic and societal burden of DKD. In the United States, the mean annual all-cause healthcare costs per insured patient with DKD range from about $30,000 in early stages to approximately $119,000 in more advanced stages [3]. These trends underscore the urgent need to elucidate the cellular and molecular mechanisms by which chronic metabolic and hemodynamic stressors promote renal injury, thereby informing the development of targeted therapeutic strategies to prevent or reverse disease progression.

DKD involves dysfunction across multiple renal cell types, including podocytes, glomerular endothelial cells, mesangial cells, and tubular epithelial cells. Under physiological conditions, podocytes and glomerular endothelial cells form and maintain the glomerular filtration barrier; mesangial cells provide structural support and regulate glomerular contraction, and tubular epithelial cells reabsorb solutes, nutrients, and water from the filtrate. Dynamic intraglomerular and glomerulotubular feedback mechanisms further integrate filtration, reabsorption, and signaling processes to preserve renal homeostasis [4]. However, in DKD, cell-specific dysfunction initiates maladaptive signaling cascades. Pathological intercellular crosstalk promotes the release of injurious factors, such as microRNAs and transforming growth factor beta (TGF-β), that propagate injury across renal compartments. These signals can lead to podocyte death, mesangial cell proliferation, and increased permeability of the glomerular filtration barrier [5]. In podocytes, DKD manifests as loss of slit diaphragm proteins, cytoskeletal remodeling, detachment, and foot process effacement [6]. Notably, a seminal paper by Wharram et al. showed that graded podocyte loss in human diphtheria toxin receptor-transgenic rats, with mesangial expansion and transient proteinuria evident even within the first ~ 20% depletion, leads to progressively more severe glomerular lesions, with greater loss (> 20 to 40%) promoting focal segmental glomerulosclerosis and > 40% loss leading to global glomerulosclerosis that parallels the glomerular injury observed in diabetic glomerulosclerosis [7]. In parallel, human and experimental DKD studies demonstrate that reduced podocyte number and increased podocyte detachment or urinary podocyte markers are associated with albuminuria and predict nephropathy progression, supporting a central role for podocyte loss in DKD pathogenesis [8–10]. Meanwhile, glomerular endothelial cells exhibit glycocalyx loss and disruption of fenestration [11, 12], and mesangial cells contribute to matrix accumulation and mesangial expansion [13]. Tubular cells, particularly in the proximal tubule, show increased sodium-glucose reabsorption, hypoxia, and albumin overload [14, 15]. Collectively, these multidimensional, compartment-specific alterations create a permissive environment for the progression of inflammation, injury, and fibrosis.

**Fig. 1:**
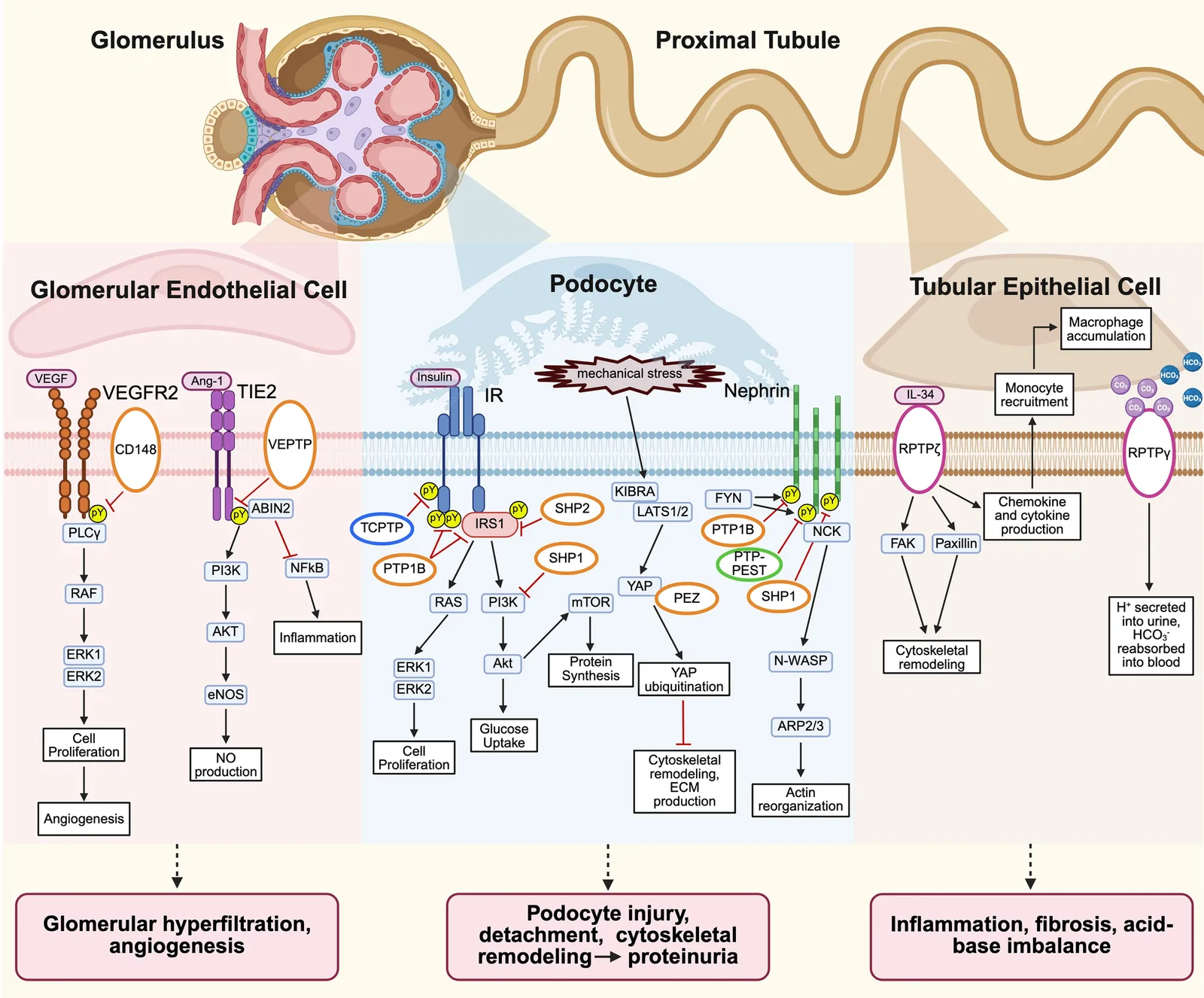
PTP-regulated signaling pathways in renal cells in DKD. Schematic overview of signaling pathways modulated by classical PTPs across major renal cell types implicated in DKD. Receptor-type PTPs are depicted as vertical ovals, whereas non-receptor-type PTPs are depicted as horizontal ovals. PTPs ranked as “+++” in Table 1 have an orange border, those ranked as “++” have a blue border, “+” have a pink border, and “−” have a green border. The pathways illustrated are representative rather than comprehensive; some connecting lines represent multistep signaling cascades involving intermediate targets that are not depicted, and some PTP-regulated signaling networks operate across more than one renal cell type but are shown here in a simplified manner for conceptual clarity, based on where the preponderance of available evidence has been reported. In glomerular endothelial cells (left), the PTPs CD148 and VEPTP dephosphorylate VEGFR2 and TIE2, respectively, downstream of VEGF and angiopoietin-1 (Ang-1), modulating angiogenesis, nitric oxide (NO) production, and inflammation, leading to glomerular hyperfiltration and pathological angiogenesis. In podocytes (center), insulin signaling is tightly regulated by PTPs, including PTP1B, TCPTP, SHP1, and SHP2, which affect IR and IR substrate 1 (IRS1) phosphorylation, and downstream pathways that control cell growth, glucose uptake, and protein synthesis. Nephrin phosphorylation, mediated by PTP1B, SHP1, and PTP-PEST, regulates actin cytoskeletal organization, while mechanical stress-responsive pathways, involving kidney and brain protein (KIBRA), large tumor suppressor kinases 1 and 2 (LATS1/2), yes-associated protein (YAP), and PEZ, further integrate cytoskeletal remodeling and extracellular matrix production, linking dysregulated signaling to podocyte injury, detachment, and proteinuria. In tubular epithelial cells (right), RPTPζ participates in inflammatory and structural responses, including interleukin-34 (IL-34)-mediated cytoskeletal remodeling and macrophage accumulation. In contrast, RPTPγ modulates acid–base handling, collectively influencing inflammation, fibrosis, and acid–base balance. This figure was created using BioRender (https://biorender.com/)

A congregation of injurious pathways intersects to initiate and propagate tissue injury in DKD. Central to disease progression is the dysregulation of phosphorylation-dependent signaling networks across several interconnected cascades, including insulin signaling, cytoskeletal organization, inflammation, and oxidative stress (Fig. 1) [16]. Podocytes and other renal cells express the insulin receptor (IR) and canonical signaling proteins, such as insulin receptor substrates 1 and 2 (IRS1/2), phosphatidylinositol-3 kinase (PI3K), and Akt serine/threonine kinase (Akt) [17]. In DKD, podocyte insulin resistance impairs PI3K/Akt and mitogen-activated protein kinase (MAPK) pathways, disrupting cell survival and cytoskeletal stability. Podocyte insulin signaling appears critical for maintaining cytoskeletal integrity, as complete deletion of the IR in podocytes destabilizes actin dynamics, contributing to foot process effacement and albuminuria [18]. This process is mediated in part through nephrin, a critical slit diaphragm protein important for insulin-stimulated actin remodeling in podocytes [19]. Nephrin and other cytoskeletal components such as vimentin [20] and α-actinin-4 [21] are regulated by phosphorylation, and dysregulation of these nodes can destabilize actin networks, contributing to effacement, detachment, and progressive proteinuria. Inflammatory cascades are also tightly tuned by phosphorylation. Hyperglycemia-associated renin–angiotensin–aldosterone system (RAAS) activation, advanced glycation end products-receptor for advanced glycation end products (AGE-RAGE) signaling, and cytokine release stimulate pro-inflammatory pathways in glomerular and tubular cells. The cellular response to these stimuli is modulated by phosphorylation-dependent mechanisms that involve janus kinase/signal transducer and activator of transcription (JAK/STAT) and nuclear factor kappa B (NFκB) signaling [22–24]. Moreover, oxidative stress intersects with both insulin and inflammatory signaling: high glucose and impaired insulin signaling increase mitochondrial and NADPH oxidase-derived reactive oxygen species (ROS), which can damage cytoskeletal proteins and trigger apoptosis [25]. Together, impaired insulin signaling, cytoskeletal disruption, inflammation, and oxidative stress converge on aberrant phosphorylation signaling, positioning phosphorylation-dependent network dysfunction as a central driver of DKD progression.

Tyrosine phosphorylation is a critical and transient post-translational modification modulated by the coordinated action of protein tyrosine kinases (PTKs) and protein tyrosine phosphatases (PTPs) [26]. PTKs generally promote signal transduction through phosphorylation, whereas PTPs typically attenuate, but in some contexts facilitate, signaling by dephosphorylation. The human genome encodes over 100 PTP genes, including 38 classical PTPs, defined by a shared architecture and selective targeting of phospho-tyrosine residues (Table 1) [27–29]. Of the classical PTPs, there are 21 receptor-type and 17 non-receptor-type enzymes. Several classical PTPs have been directly implicated in DKD pathogenesis or related renal and metabolic disorders, while others modulate signaling pathways relevant to DKD progression [26, 30, 31]. Conversely, some appear to have limited or no direct evidence to date in DKD pathophysiology. Elucidating the contributions of the classical PTPs to DKD may reveal novel therapeutic targets and offer insight into the integrative signaling mechanisms underlying the disease. This review synthesizes current knowledge on classical PTPs in DKD, highlighting their mechanistic roles in disease development and progression, and considers how pharmacological targeting of PTPs may be integrated into existing and emerging treatment strategies.

**Table 1:** The 38 classical PTPs and their reported evidence in DKD

Sub-type	Gene	Protein	Experimental models			Disease context			Total evidence
			Cell	Animal	Human	DKD	DM	Renal injury	
Receptor-type	*PTPRA*	RPTP⍺, LRP	x		x		x		+
	*PTPRB*	VEPTP	x	x	x	x			+ + +
	*PTPRC*	CD45, LCA			x	x		x	+ +
	*PTPRD*	RPTPδ	x		x		x		+
	*PTPRE*	RPTPε	x	x	x		x		+
	*PTPRF*	LAR	x	x			x		+
	*PTPRG*	RPTPγ	x		x			x	+
	*PTPRH*	SAP1	x						−
	*PTPRJ*	CD148, DEP1, RPTPη	x	x	x	x			+ + +
	*PTPRK*	RPTPκ			x		x		+
	*PTPRM*	RPTPμ	x					x	+
	*PTPRN*	IA-2, Islet cell antigen 512			x	x	x	x	+ +
	*PTPRN2*	IA-2β, phogrin			x	x	x	x	+ +
	*PTPRO*	GLEPP1, PTP-U2, PTPOC, PTPROτ	x	x	x	x			+ + +
	*PTPRQ*	rPTP-GMC1, PTPS31	x	x	x			x	+
	*PTPRR*	PTP-SL, PCPTP, PTPBR7, PC12-PTP1			x			x	+
	*PTPRS*	RPTPσ		x	x		x		+
	*PTPRT*	RPTPρ		x	x		x		+
	*PTPRU*	PTP-U1, PCP-2, PTPRomicron			x		x		+
	*PTPRV*	OST-PTP							−
	*PTPRZ*	RPTPζ	x	x				x	+
Non-receptor type	*PTPN1*	PTP1B	x	x	x	x			+ + +
	*PTPN2*	TCPTP, MPTP, PTP-S	x	x	x	x	x		+ + +
	*PTPN3*	PTPH1	x		x		x		+
	*PTPN4*	PTP-MEG1, TEP	x				x		+
	*PTPN5*	STEP		x			x		+
	*PTPN6*	SHP1, PTP1C, SH-PTP1, HCP	x	x	x	x			+ + +
	*PTPN7*	HePTP, LCPTP			x		x		+
	*PTPN9*	PTP-MEG2	x	x			x		+
	*PTPN11*	SHP2, SH-PTP2, Syp, PTP1D, PTP2C, SH-PTP3	x	x	x	x			+ + +
	*PTPN12*	PTP-PEST, PTP-P19, PTPG1	x						−
	*PTPN13*	PTP-BAS, FAP-1, PTP1E, RIP, PTPL1, PTP-BL	x						−
	*PTPN14*	PTP36, PEZ, PTPD2	x	x	x	x			+ + +
	*PTPN18*	PTP-HSCF, BDP	x				x		+
	*PTPN20*	TypPTP, hPTPN20a							−
	*PTPN21*	PTPD1, PTP2E, PTP-RL10	x						−
	*PTPN22*	LYP, PEP	x		x		x		+
	*PTPN23*	HD-PTP, PTP-TD14	x						−

Sub-type indicates receptor-type (transmembrane) or non-receptor type (intracellular) PTPs. Experimental Model denotes the systems in which evidence has been reported: Cells (in vitro cell culture), Animals (in vivo animal models), and Humans (clinical studies or human-derived samples); "x" indicates available evidence. Disease Context includes DKD (diabetic nephropathy), DM (diabetes or glucose or insulin metabolism without specific renal focus), and Renal Injury (kidney damage in non-diabetic settings). Total Evidence provides a qualitative assessment of the overall evidence supporting each PTP in DKD. The score reflects multiple considerations, including the breadth of experimental systems, the degree of mechanistic validation, and the relevance of the findings to DKD, and is not intended to represent a numerical algorithm. +++, strong evidence across multiple experimental models with substantial support in the DKD context; ++, moderate evidence with support from multiple studies or experimental systems and relevance to DKD; + , emerging or preliminary evidence, primarily from limited studies or in the contexts of DM and/or renal injury; − , available studies report no association with DKD or evidence is limited and outside the listed disease contexts

## PTPs in DKD

PTPs were first implicated in insulin signaling and, thus, in diabetes in the late 1980s and early 1990s, with studies showing that the activated IR undergoes tyrosine dephosphorylation that attenuates receptor signaling [32, 33]. The classical PTPs, *PTPN1* (PTP1B) and *PTPRF* (LAR), were among the first to be identified as negative regulators of insulin signaling by dephosphorylating the IR and/or downstream insulin signaling proteins [34, 35]. Around this period, the potent broad-spectrum phosphatase inhibitor, vanadate, was found to exert “insulin-like” effects in experimental models [36] and was later extended to studies of DKD to examine its impact on renal cells [37]. These seminal discoveries provided a mechanistic rationale for investigating the roles of PTPs in DKD pathogenesis. Since then, many classical PTPs have been examined in the context of DKD, diabetes, and renal physiology. In the sections that follow, we categorize these PTPs based on the strength and specificity of experimental evidence linking them to DKD, as reflected in the evidence weighting summarized in Table 1: those directly established in DKD, those indirectly implicated in disease-relevant contexts, and those with limited or no direct evidence to date.

### PTPs established in DKD

*PTPN1* (PTP1B) directly modulates insulin signaling in large part by dephosphorylating the IR (Fig. 1) [38–42]. In immortalized murine podocyte cells, PTP1B knockdown reduces the migratory phenotype and stabilizes the cytoskeleton [21, 43]. Additionally, PTP1B knockdown in immortalized murine podocytes subjected to high glucose preserves insulin signaling [44]. Moreover, we identified α-actinin-4, a cytoskeletal constituent abundantly expressed in podocytes, as a PTP1B substrate in insulin-stimulated podocytes and linked its function to podocyte motility [21]. Complementing this, podocyte-specific PTP1B overexpression in mice promotes cytoskeletal disorganization and enhances motility [43]. In rodent models of diabetic renal injury, PTP1B is upregulated in glomeruli [45], and global PTP1B genetic deletion markedly attenuates glomerular injury, ameliorates endoplasmic reticulum (ER) stress, and reduces albuminuria in endothelial nitric oxide synthase (eNOS)-deficient diabetic mice, without major alterations in systemic blood glucose or blood pressure [46]. In vivo podocyte-specific genetic deletion or pharmacological inhibition of PTP1B reduces albuminuria, preserves podocyte architecture, and attenuates glomerular basement membrane (GBM) thickening (Table 2) [43, 44]. In keeping with observations in animal models, PTP1B expression has been reported to be elevated in glomerular biopsies from humans with DKD [47]. Mechanistically, PTP1B overexpression may contribute to DKD pathogenesis by antagonizing insulin signaling [42], exacerbating ER stress [48], activating focal adhesion kinase (FAK) signaling [43], and augmenting the expression of inflammatory cytokines [47, 49].

**Table 2:** Genetic modulation of classical PTPs in murine models of renal injury

PTP	Cell type	Disease model, genetic background	Effects on Key Indices of Renal Function	References
*PTPRB*	Whole body inducible KO	Ins2, Akita;RenAAV	↑ endothelial cells ↑ GFR ↑ podocytes ↓ albuminuria ↓ fibrosis ↓ glomerulosclerosis	[50]
*PTPRO*	Whole body KO	Uninephrectomy, C57BL/6J	↑ blood pressure ~ albuminuria ↓ GFR	[51]
*PTPRZ*	Whole body KO	Ischemia/reperfusion injury, C57BL/6J	↑ TECs ↑ tubular regeneration ↓ fibrosis	[52]
*PTPN1*	Podocyte-specific KO	Adriamycin (12.5 mg/kg) or lipopolysaccharide (200 μg), ICR × 129 x C57BL6J	↓ albuminuria	[43]
		High-fat-diet or STZ (160 mg/kg × 1 day), C57BL/6J	~ serum creatinine ↓ albuminuria ↓ fibrosis ↓ glomerulosclerosis ↓ inflammation	[44]
	Whole body KO	eNOS KO, C57BL/6J	~ serum creatinine ↓ albuminuria	[46]
*PTPN6*	Podocyte-specific KO	Ins2, Akita	↑ podocytes ↓ albuminuria ↓ GFR (normalized) ↓ mesangial matrix expansion	[53]
*PTPN11*	Podocyte-specific KO	Lipopolysaccharide (16 µg/g), 129 Sv/J x C57Bl/6J	↓ albuminuria ↓ BUN ↓ inflammation	[54]
		High-fat-diet, STZ (160 mg/kg × 1 day), or STZ (50 mg/kg × 5 days), 129 Sv/J x C57Bl/6J	↓ albuminuria ↓ blood pressure ↓ BUN ↓ fibrosis ↓ inflammation	[55]
	Macrophage-specific KO	STZ (100 mg/kg × 1 day), C57BL/6J	↓ BUN ↓ fibrosis ↓ glomerulosclerosis ↓ inflammation ↓ mesangial matrix expansion ↓ serum creatinine	[56]
*PTPN14*	Whole body KO	STZ (55 mg/kg × 5 days), C57BL/6J	↑ podocytes ↓ albuminuria ↓ fibrosis ↓ glomerulosclerosis	[57]

Included PTPs are those in which a classical PTP was genetically modulated in a murine model of renal injury. “Cell type” refers to the site of genetic modulation, including whole-body knockout (KO) and cell-specific models. “Disease Model, Genetic Background” denotes the kidney injury model and mouse strain used in the associated study. Effects on key indices of renal function are indicated if reported in the respective study, including albuminuria, blood pressure, BUN, endothelial cells, fibrosis, GFR, glomerulosclerosis, inflammation, mesangial matrix expansion, podocytes, serum creatinine, TECs, and tubular regeneration. Upward and downward arrows (↑/↓) indicate an increase or decrease in the associated marker, respectively, while a tilde ( ~) indicates no change

*PTPN11* (SHP2) has emerged as a modulator of DKD pathogenesis through its roles in podocyte signaling, inflammation, and growth factor cascades (Fig. 1). In E11 murine podocytes, SHP2 protein expression increases under culture in high glucose, accompanied by increased ER stress, NFκB activation, and cell motility, whereas SHP2 knockdown prevents these changes [55]. The increased motility may be linked to SHP2-mediated cytoskeletal remodeling, as vimentin, an intermediate filament critical for podocyte migration and cytoskeletal remodeling, has been implicated in SHP2-modulated growth factor signaling in non-renal cell lines [20]. In diabetic mouse models, elevated SHP2 phosphorylation and protein expression are observed in macrophages, with increased SHP2 expression also detected in glomeruli and podocytes [55, 56]. Notably, podocyte-specific genetic deletion or pharmacological inhibition of SHP2 in rodent models of hyperglycemia or kidney injury preserves renal function (Table 2) [54, 55, 58]. These animals exhibit lower albuminuria and blood urea nitrogen (BUN) levels, as well as protection against glomerular structural changes. Moreover, in a leptin receptor-deficient model of diabetes, pharmacological inhibition of SHP2 demonstrates renoprotective effects including reduced albuminuria and improved histopathology [56]. Moreover, immunohistochemical analysis demonstrates elevated phosphorylated SHP2 in renal biopsies from patients with DKD at pY542, a major in vivo phospho-site and key regulatory site for SHP2 activation [56, 59]. In other kidney diseases, including minimal-change nephrosis and membranous nephropathy, increased SHP2 phosphorylation has also been observed at pY542 [58]. Collectively, these studies indicate that inactivation of SHP2 may mitigate DKD progression, at least in part, by attenuating ER stress and inflammation, while improving insulin signaling and cytoskeletal stability.

*PTPN6* (SHP1) contributes to DKD pathogenesis through its regulation of podocyte structure and insulin signaling (Fig. 1). Podocytes cultured in high glucose show elevated *PTPN6* mRNA and protein expression, which disrupts insulin signaling and promotes apoptosis, effects mitigated by overexpression of dominant-negative SHP1 [60]. Functionally, SHP1 modulates insulin signaling by dephosphorylating the p85 regulatory subunit of PI3K [61], a central downstream signaling intermediate in insulin signaling, and nephrin, a key mediator of podocyte architecture and filtration barrier integrity [62]. In diabetic mouse models, SHP1 is upregulated in the renal cortex and specifically enriched in podocytes [60, 63]. Podocyte-specific genetic deletion of SHP1 in a mouse model of DKD reduces albuminuria, attenuates foot process effacement and GBM thickening, and prevents changes in mRNA expression of actin cytoskeleton signaling and remodeling markers (Table 2) [53]. Moreover, its expression is elevated in the glomeruli of patients with diabetes and DKD [53, 64].

*PTPRB* (VEPTP) is an endothelial phosphatase that directly dephosphorylates TEK receptor tyrosine kinase (TIE2, Fig. 1) [65, 66]. It is highly expressed in glomerular capillaries, arterioles, and interlobular vessels and, in the adult kidney, is largely restricted to endothelial cells [50]. In primary human glomerular endothelial cells, hyperglycemia, osmotic stress, and hypoxia increase VEPTP expression [50], and in diabetic mouse models, VEPTP expression is elevated in the renal cortex [67]. Similarly, in an acute kidney injury (AKI) ischemia/reperfusion injury model, VEPTP expression increases in renal endothelial cells and is associated with reduced TIE2 signaling, whereas *PTPRB* genetic deletion preserves endothelial integrity, limits inflammation, and improves renal function [68]. Inducible VEPTP genetic deletion in a diabetic mouse model improves GFR, reduces glomerular matrix accumulation and sclerosis, and preserves podocyte and slit diaphragm integrity (Table 2) [50]. However, VEPTP inhibition with the extracellular domain-targeting antibody mAb 109.1 alone does not significantly improve albuminuria or BUN in experimental DKD [67], suggesting that while endothelial protection enhances renal hemodynamics and structural resilience, it may be insufficient to fully correct filtration barrier dysfunction in DKD.

*PTPRO* (GLEPP1) is selectively expressed on the apical surface of podocytes, and mice with global GLEPP1 genetic deletion exhibit altered podocyte morphology, appearing ameboid rather than octopoid, with short and broad foot processes [51, 69]. These structural abnormalities are accompanied by altered vimentin distribution and reduced nephrin content, leading to a reduced filtration area, which corresponds with a measurable decline in GFR at baseline and following uninephrectomy [51]. Further, these GLEPP1-deficient mice exhibit higher blood pressure than controls, despite no difference in proteinuria (Table 2) [51]. Moreover, in the puromycin aminonucleoside nephrosis model of podocyte injury in rats, GLEPP1 expression is transiently reduced early after injury and subsequently recovers, remaining completely absent only in sclerosed glomeruli [70, 71]. Downregulation of GLEPP1 has also been reported in human DKD [72], where its expression is diminished in glomeruli with mesangial expansion and is absent in sclerosed glomeruli [73, 74]. While its involvement in podocyte insulin signaling remains to be defined, the influence of GLEPP1 on cytoskeletal organization and nephrin-dependent barrier integrity indicates that it may contribute to the structural and functional decline observed in DKD.

*PTPRJ* (CD148) modulates epithelial and endothelial signaling by dephosphorylating key growth factor receptors, including platelet-derived growth factor receptor beta (PDGFRβ), epidermal growth factor receptor (EGFR), and vascular endothelial growth factor receptor 2 (VEGFR2) (Fig. 1) [75–77]. In vitro, CD148 overexpression in kidney fibroblast cells (NRK-49F) inhibits TGF-β-induced proliferation and reduces the expression of fibrosis markers through the dephosphorylation of PDGFRβ [78]. Moreover, in murine models of diabetes, podocyte-specific genetic deletion or pharmacological inhibition of EGFR attenuates albuminuria and limits podocyte loss [79, 80]. Consistent with these findings, treatment with an agonistic anti-CD148 antibody in a diabetic mouse model enhanced CD148 activity and subsequently attenuated albuminuria and mesangial matrix expansion, suppressed glomerular markers of fibrosis and macrophage infiltration, and preserved nephrin expression and podocyte number [81]. In addition to PDGFRβ and EGFR signaling, CD148 also regulates VEGFR2, the primary mediator of VEGF-dependent angiogenesis. CD148 modulates this process by dephosphorylating VEGFR2 to inhibit excessive endothelial cell proliferation and migration while simultaneously activating proto-oncogene tyrosine-protein kinase Src (SRC)/Akt signaling to ensure proper cell survival and stable capillary formation [76]. Excessive VEGF signaling promotes pathological angiogenesis and vascular permeability in diabetes and other chronic diseases [82, 83]. In patients with diabetes, urinary VEGF levels correlate positively with proteinuria severity, while plasma VEGF levels are elevated only in overt proteinuria [84]. These findings suggest that urinary VEGF may be a more sensitive indicator of progressive renal injury in DKD and underscore the relevance of VEGF signaling dysregulation in DKD. These data suggest that CD148-mediated modulation of PDGFRβ, EGFR, and VEGFR2 signaling helps preserve podocyte integrity and endothelial homeostasis, thereby limiting albuminuria and structural progression in DKD.

*PTPN14* (PEZ) modulates the Hippo pathway through its interaction with YAP; Fig. 1), a transcriptional co-activator that regulates cellular proliferation and apoptosis [85]. Although often studied in the context of Hippo pathway dysregulation in cancer, PEZ also contributes to adherens junction dynamics, endothelial barrier function, and regulation of inflammation [86, 87]. In vitro, both PEZ mRNA and protein expression increase in response to high glucose culture [57]. Global PEZ genetic deletion in a streptozotocin (STZ)-induced diabetic mouse model mitigates renal injury, attenuating albuminuria and preserving glomerular architecture (Table 2) [57]. Functionally, PEZ deficiency suppresses glomerular markers of inflammation and fibrosis, potentially via interactions with Thyroid Receptor-Interacting Protein 6 (TRIP6) [57]. In glomeruli of diabetic mice and in kidneys of patients with DKD, *PTPN14* mRNA and protein levels are elevated, with expression detected in podocytes [57]. In DKD, PEZ likely promotes glomerular injury mainly through TRIP6-mediated inflammatory and fibrotic signaling in podocytes.

*PTPN2* (TCPTP), a close homolog of PTP1B, dephosphorylates the IR in a non-redundant manner (Fig. 1) [88–90]. In vitro, high glucose suppresses *PTPN2* expression in murine mesangial and tubular epithelial cells, and in a mouse model of diabetes and hyperlipidemia (STZ-induced diabetes on an ApoE^−/−^ background), adenoviral gene therapy for systemic overexpression of *PTPN2* ameliorates albuminuria, lowers serum creatinine and BUN, and improves glomerular, tubular, and interstitial morphology (Table 2) [91]. However, because this intervention also attenuates hyperglycemia and dyslipidemia, the renal improvements may result from improved metabolic control rather than direct kidney-specific *PTPN2* effects. In patients with early-stage DKD (stages 1–2), TCPTP expression in serum and peripheral blood mononuclear cells was reduced, with the lowest levels observed in patients with macroalbuminuria [92]. Mechanistically, TCPTP appears to mitigate renal injury associated with DKD, at least in part, by ameliorating inflammation, fibrosis, and STAT-dependent signaling [91].

### PTPs implicated in DKD

*PTPRG* (RPTPγ) is expressed in renal cells including podocytes, glomerular endothelial cells, fibroblasts, and proximal tubular epithelial cells (PTECs), where its expression is downregulated under oxidative stress via miR-141-mediated suppression [93]. In PTECs, RPTPγ localizes predominantly to the basolateral membrane and functions as an extracellular CO₂/HCO₃⁻ sensor important for renal pH homeostasis (Fig. 1) [94]. Moreover, RPTPγ was identified in a serum proteomics study as one of several candidate serum proteins that exhibited increased abundance during DKD progression [95].

*PTPRC* (CD45) is highly expressed on all nucleated hematopoietic cells and is often used as a canonical pan-leukocyte marker [96, 97]. In diabetic mice, macrophage infiltration correlates with declining renal function, and immune-mediated inflammation contributes to renal fibrosis [98, 99]. CD45 was identified as a highly differentially expressed gene (DEG) associated with kidney fibrosis across multiple chronic kidney diseases, including DKD, with expression levels inversely correlating with GFR [100, 101]. It demonstrated the highest degree score in the common fibrosis protein–protein interaction network, supporting the significant contributions of immune cell-associated signaling to renal fibrosis across chronic kidney diseases.

*PTPRZ* (RPTPζ) functions as a receptor for IL-34 in inflamed renal tissue and contributes to macrophage accumulation (Fig. 1), which in turn promotes macrophage-mediated tubular epithelial cell (TEC) apoptosis in AKI [52, 102]. Preliminary evidence shows that in a mouse model of DKD, IL-34 expression is elevated and IL-34 genetic deletion mitigates renal injury and macrophage infiltration [103]. Moreover, during kidney injury in a lupus nephritis mouse model, both IL-34 and RPTPζ expression are increased [104]. In an AKI-to-CKD ischemia/reperfusion injury model, global *PTPRZ* genetic deletion preserves TEC integrity, reduces apoptosis, enhances tubular regeneration, and confers protection against fibrosis (Table 2) [52].

### PTPs implicated in DM

*PTPN5* (STEP) selectively binds and dephosphorylates specific members of the MAPK family. It is highly expressed in the nervous system [105] and has been detected in the renal cortex [106]. In a diet-induced murine model of obesity, *PTPN5* expression is markedly increased in the renal cortex, with no corresponding changes in *PTPN5* expression in the liver or heart [107]. Moreover, in the *db/db* mouse model of DM, treatment with *Apostichopus japonicus enzymatic hydrolysate*, a nutraceutical, significantly downregulated *PTPN5* expression in renal tissue while improving glycemic control, dyslipidemia, and kidney index [108]. These findings implicate STEP as a potential contributor to metabolic and renal dysfunction in diabetes and suggest that it may represent a target for therapeutic intervention.

*PTPRD* (RPTPδ) is most highly expressed in the brain, followed by the kidney [109]. RPTPδ knockdown in vitro decreases IR expression, implicating this phosphatase as a potential modulator of insulin signaling [110]. Certain RPTPδ variants have been linked to T2DM risk [111], with specific associations observed in a rural Han Chinese population [112]. In addition, *PTPRD* expression levels are reduced in T2DM and correlate with disease duration [110]. The CC genotype of *PTPRD* rs56407701 is associated with suppressed RPTPδ expression and elevated HbA1c and fasting plasma glucose in mothers with gestational diabetes [113]. However, other *PTPRD* variants have been associated with a decreased risk of gestational diabetes [114].

*PTPRU* (PTP-U1) contains two pseudophosphatase domains that render it catalytically inactive due to several structural rearrangements that lead to the destabilization of the active site pocket and block the catalytic cysteine [115]. Despite this, it influences signaling by acting as a scaffold and may compete with active phosphatases for substrate binding. It localizes to adherens junctions, and evidence suggests it interacts with β-catenin, affecting its transcriptional activity and contributing to epithelial integrity [116]. One transcriptomic study identified *PTPRU* as a DEG associated with glucocorticoid signaling in skeletal muscle from patients with T2DM [117]. Additionally, a US patent (7897583B2) proposes antisense-based approaches targeting *PTPRU* as a potential therapeutic strategy for diabetes treatment [118].

*PTPN9* (PTP-MEG2) has been proposed as a potential therapeutic target for T2DM. In primary rat hepatocytes, PTP-MEG2 modulates the phosphorylation of the IR at key activation loop tyrosine residues (1162/1163), and PTP-MEG2 overexpression blocks insulin-mediated repression of phosphoenolpyruvate carboxykinase (PEPCK) [119]. In vivo, hepatic overexpression of PTP-MEG2 impairs glucose tolerance and induces insulin resistance, whereas adenoviral-mediated hepatic depletion of *PTPN9* in a diabetic mouse model improves glucose clearance and insulin sensitivity [119]. Small-molecule inhibitors derived from *Gingko biloba* and phloridzin, found in apple tree bark and leaves, have been shown to inhibit PTP-MEG2 activity in vitro, with associated increases in adenosine monophosphate-activated protein kinase (AMPK) or Akt phosphorylation and improved glucose uptake [120, 121].

*PTPN3* (PTPH1) dysregulation has been implicated in the pathophysiology of several cancers, including renal cell carcinoma. In tumor cells, PTPH1 inhibits the PI3K/Akt pathway [122] and enhances TGF‐β signaling independently of its phosphatase activity [123]. Emerging evidence also suggests a link between PTPH1 and metabolic dysfunction. Its expression is reduced in adipose and muscle tissue of individuals with insulin resistance compared to insulin-sensitive controls, matched for body mass index [124], and in some cohorts of patients with T2DM, certain *PTPN3* single-nucleotide polymorphisms (SNPs) occur at higher allelic frequency [125].

*PTPN22* (LYP) is expressed in multiple immune cell types and is a key negative regulator of T-cell receptor signaling [126]. The *PTPN22* R620W variant is strongly associated with increased susceptibility to several autoimmune diseases, including type 1 diabetes (T1DM) [127, 128]. The same variant has also been evaluated in genetic association studies of T2DM-related metabolic traits such as glycemic regulation [129], and *PTPN22* copy number gain has been reported in some cohorts to be associated with increased risk for T2DM [130]. Epigenetic regulation by DNA methyltransferase 1 (DNMT1) influences *PTPN22* expression, and in cystic renal epithelial cell models, *PTPN22* has been identified as a mediator through which DNMT1 modulates STAT3, ERK, and mTOR/S6 ribosomal protein signaling, implicating this phosphatase in inflammatory and growth-related pathways [131].

*PTPN7* (HePTP) is a hematopoietic PTP that specifically binds members of the MAPK family, including ERK and p38, and negatively regulates their activity [132]. Some transcriptomic studies report increased HePTP expression in monocytes from patients with T1DM or T2DM [133, 134]. Collectively, these studies suggest a potential role for HePTP in diabetes-associated immune dysregulation.

*PTPRN* (IA-2) and *PTPRN2* (IA-2β) are enzymatically inactive phosphatases and major autoantigens in T1DM [135, 136]. Both proteins are expressed in pancreatic islets and modulate insulin content and secretion in β-cells [137, 138]. In addition, IA-2 and possibly IA-2β have been implicated in the regulation of β-cell growth and proliferation through STAT-dependent signaling [139, 140]. In the kidney, both proteins are associated with sympathetic nerve terminals along afferent arterioles, where they contribute to the maintenance of normal renin levels through the β-adrenergic pathway but do not appear to affect GFR [141]. Moreover, *PTPRN* family members have been identified in genetic and epigenetic studies of CKD and DKD, suggesting a possible association with kidney disease risk [142, 143].

*PTPRK* (RPTPκ) plays a role in thymocyte and early thymus development, which may influence immune tolerance and autoimmunity [144, 145]. Variants in the *PTPRK-THEMIS* locus have been associated with T1DM in children diagnosed at a young age, and functional studies suggest that *THEMIS* expression is a key mediator [146, 147]. Beyond its immunologic role, RPTPκ has also been linked to Alzheimer’s Disease [148].

*PTPRE* (RPTPε) has two major isoforms, one anchored at the cell membrane (memRPTPε) and a cytosolic form (cytRPTPε), the latter of which exhibits partial nuclear localization [149, 150]. Both forms have been shown to interact with the IR. In primary hepatocytes, memRPTPε dephosphorylates the IR at key tyrosine residues, thereby reducing downstream Akt and ERK activation [151]. In L6 skeletal muscle cells, insulin promotes cytRPTPε association with the IR followed by co-internalization; cytRPTPε knockdown enhances insulin-induced IR and IRS-1 phosphorylation and increases glucose uptake, while overexpression suppresses these effects [152]. Beyond insulin signaling, RPTPε is upregulated in vascular smooth muscle cells of salt-sensitive hypertensive mice, where it contributes to impaired vasomotor function [153]. Also, RPTPε has been reported as a DEG in microvascular complications of diabetes, including diabetic nephropathy, suggesting potentially broader relevance to diabetes-related vascular dysfunction [154, 155].

### PTPs in relevant signaling pathways

*PTPN12* (PTP-PEST) is expressed in cultured mouse podocytes and negatively regulates Fyn-dependent nephrin phosphorylation [156]. Although its protein expression level remains largely unchanged in vitro in models of podocyte foot process effacement [157], PTP-PEST is a key regulator that promotes cell migration and focal adhesions turnover in response to integrin stimulation [158].

*PTPN18* (PTP-HSCF) is highly expressed in hematopoietic progenitor cells, where it promotes and sustains erythropoietin (EPO)-induced phosphorylation of ERK1/2, Akt, STAT5 and JAK2 [159]. PTP-HSCF contributes to metabolic reprogramming in cancer, as its inactivation in vitro reduces glucose consumption and expression of glycolysis-related proteins [160]. Additionally, PTP-HSCF expression has been associated with metabolic syndrome traits, including elevated fasting insulin, suggesting a context-dependent role in metabolic regulation that may influence susceptibility to insulin resistance and T2DM [161].

*PTPRF* (LAR) is widely expressed and directly dephosphorylates the IR at key activation-loop tyrosine residues within the kinase domain of the β-subunit [35, 162, 163]. Consistent with a negative regulatory role, LAR knockdown in vitro attenuates palmitate-induced insulin resistance in C2C12 myotubes [164]. In vivo, transgenic mice overexpressing human LAR in muscle exhibit whole-body insulin resistance with elevated fasting insulin and reduced glucose infusion rate during hyperinsulinemic-euglycemic clamp studies [165]. Notably, within skeletal muscle of these mice, insulin-stimulated IR and IRS1 phosphorylation remain unchanged, whereas IRS2 phosphorylation is markedly reduced, indicating substrate‑ and tissue‑selective actions of LAR on insulin signaling. Moreover, in adipose tissue from obese patients, LAR expression is increased, and immunodepletion of LAR from adipose tissue homogenates normalized the elevated phosphatase activity toward the IR, indicating that increased LAR abundance accounted for the enhanced receptor dephosphorylation observed in obese subjects[166].

*PTPRM* (RPTPμ) is expressed in the endothelial and epithelial compartments of the kidney, including arterial and glomerular vessels [167, 168]. It contributes to epithelial cell–cell adhesion and adherens junction stability [169]. RPTPμ dephosphorylates phosphatidylinositol-4-phosphate 5-kinase type 1 gamma (PIPKIγ) and prevents its activation of integrin αvβ3 at cell–cell adhesion sites in Madin-Darby canine kidney cells [170]. In addition to these structural roles, *PTPRM* knockdown in cystic renal epithelial cell models increases phosphorylation of STAT3, ERK, and the S6 ribosomal protein, consistent with a role in modulating MAPK, mTOR, and JAK/STAT signaling [131].

*PTPRS* (RPTPσ) modulates growth factor signaling, in part, by dephosphorylating the EGFR and other RTKs in specific cellular contexts [171]. Mice with whole-body genetic deletion of RPTPσ present with fasting hypoglycemia and are more sensitive to exogenous insulin, and this phenotype is substantially rescued by a low dose of human growth hormone (GH) administration in keeping with a neuroendocrine GH/insulin-like growth factor 1 (IGF-1) deficit [172, 173]. In humans, three SNPs in the *PTPRS* gene show significant allelic association with T2DM when compared with normal glucose-tolerant controls [174]. Beyond metabolic regulation, in subcutaneous small arteries from hypertensive patients with CKD, miR-338-3p expression is inversely associated with RPTPσ levels, consistent with microRNA-dependent regulatory mechanisms that may implicate RPTPσ in CKD-associated vascular pathology [175].

*PTPRT* (RPTPρ) is implicated as a negative regulator of STAT3 signaling and has been linked to the regulation of the leptin-STAT3 axis [176, 177]. Global genetic deletion of *PTPRT* in mice confers resistance to high-fat-diet-induced obesity, characterized by lower food intake, improved peripheral insulin sensitivity with lower blood glucose and insulin levels, and lower homeostatic model assessment of insulin resistance (HOMA-IR) [178]. Consistent with enhanced leptin signaling, these mice exhibit increased hypothalamic STAT3 phosphorylation, a key node in appetite and energy balance. Additionally, two specific *PTPRT* variants were among novel gene regions showing suggestive associations with fasting insulin levels in a large multi-ethnic whole-genome sequencing study [179].

*PTPN4* (PTP-MEG1) has been implicated in barrier cell behavior and cytoskeletal dynamics. At the post-transcriptional level, circRNA-*PTPN4* can increase forkhead box O3 (FOXO3) expression, resulting in upregulation of zonula occludens-1 (ZO-1) in human brain microvascular endothelial cells, consistent with regulation of blood–brain barrier permeability [180]. Furthermore, in HEK293 cells, PTP-MEG1 negatively regulates CRK proto-oncogene, adaptor protein I-mediated proliferation and wound healing migration [181]. In an insect model (Oedaleus asiaticus), FOXO family transcription factors are regulated downstream of the IR/PTP-MEG1 signaling axis, which engages the PI3K/Akt cascade. In this model, interference of *PTPN4* strongly increases the expression of IR, IRS, PI3K, PDK, and Akt and is associated with reduced expression of FOXO family transcription factors [182].

*PTPN13* (FAP-1) plays a role in growth factor signaling and has been implicated in pancreatic β-cell function. In breast cancer cells (MCF7), FAP-1 has been shown to dephosphorylate IRS1 following IGF1 stimulation, thereby attenuating IGF1 receptor-dependent activation of the IRS1/PI3K/Akt signaling cascade [183]. Concordantly, FAP-1 knockdown enhances IGF1-mediated survival signaling. In INS-1 β-cells, FAP-1 overexpression suppresses cell proliferation without altering the rise in insulin secretion triggered by various stimuli, and in response to wingless-type MMTV integration site family, member 3A (WNT3a) treatment, this overexpression prevents β-catenin accumulation [184]. Mechanistically, FAP-1 has been reported to interact with the adenomatous polyposis coli protein, which is involved in WNT signaling and can form a complex with β-catenin.

*PTPN21* (PTPD1) is involved in cellular architecture and growth factor signaling. It forms distinct complexes with actin, SRC, and FAK, and promotes cytoskeletal remodeling by modulating SRC/FAK signaling at adhesion sites [185]. In A549 cells, PTPD1 overexpression induces cell scattering and migration, and conversely, knockdown reduces cell motility [186]. Beyond structural roles, PTPD1 potentiates EGFR signaling by forming dynamic complexes with EGFR and facilitating the spatial propagation of its phosphorylated targets during early EGF signaling [187]. It is also a positive regulator of SRC-EGFR signaling, prolonging the duration and amplitude of EGFR signaling [188].

*PTPN23* (HD-PTP) regulates cell adhesion and migration. Its knockdown in endothelial cells leads to FAK hyperphosphorylation, a scattered morphology, and increased motility [189]. HD-PTP has also been shown to impart metabolic effects. Mice with a HD-PTP hypomorphic mutation exhibit marked lipodystrophy and altered lipid handling, consistent with a role for this phosphatase in endosomal sorting complexes required for transport-dependent cholesterol trafficking and homeostasis [190]. These metabolic defects are associated with reduced signaling through RAS/MAPK and PI3K/Akt pathways in white adipose tissue, evidenced by decreased phosphorylation of ERK1/2, Akt, and mTOR.

*PTPRA* (RPTP⍺) is a positive regulator of multiple Src family kinases and has been implicated in regulating cell adhesion through SRC activation [191, 192]. In vitro studies suggest that it may regulate insulin secretion [193]. Additionally, GWAS have reported suggestive links between *PTPRA* variants and fasting plasma glucose levels [194, 195].

*PTPRH* (SAP1) expression is largely restricted to the gastrointestinal tract in mice [196]. In an immortalized fibroblast cell line, SAP1 overexpression induces apoptosis and suppresses Akt and integrin-linked kinase (ILK) activation [197]. These effects are partially rescued by constitutively active forms PI3K or Akt co-expression, suggesting a role in regulating survival through the PI3K/Akt pathway.

### PTPs likely unrelated to DKD

*PTPRQ* (rPTP-GMC1) was first described in a rat model of glomerulonephritis, where its expression was transiently increased during the period of mesangial cell migration and proliferation following injury [198]. Subsequently, studies demonstrate that distinct promoters drive tissue-specific expression; the transmembrane form is expressed on the basal membrane of podocytes, while the cytoplasmic transcript predominates in mesangial cells [199]. However, rPTP-GMC1 has been most commonly associated with auditory function. To date, at least 30 *PTPRQ* variants have been reported to cause sensorineural hearing loss [200, 201]. Mouse models with *PTPRQ* mutations exhibit defective maturation of cochlear hair-bundle architecture, followed by progressive stereocilia and hair cell loss, ultimately leading to deafness [202]. Clinical case studies have similarly reported *PTPRQ* mutations in individuals with sensorineural deafness and vestibular dysfunction [203, 204].

*PTPRR* (PTP-SL) has been identified in a GWAS in a Japanese population as a candidate susceptibility gene for IgA nephropathy [205], and its druggability has been explored [206]. However, despite initial interest in its involvement in diabetes, PTP-SL mutations do not segregate with T2DM in families or across populations [207]. Separately, PTP-SL has been reported as a signature gene and risk-associated predictor for bladder cancer in transcriptomic and clinical dataset analyses [208–210].

*PTPN20* (TypPTP) is expressed in multiple tissues and localizes to sites of actin polymerization in vitro [211]. Despite limited functional characterization, TypPTP has been implicated in several cancers. Its expression is elevated in patients with triple-negative breast cancer, where it may contribute to metastatic progression [212]. TypPTP has also been identified as an innate immunity-related gene associated with *Helicobacter pylori*-related gastric cancer [213].

*PTPRV* (OST-PTP) is a pseudogene in humans but is well characterized in rodents [214]. In mice, its expression is enriched in bone and testis where it regulates the carboxylation and bioactivity of osteocalcin, a hormone implicated in systemic energy metabolism [215]. Genetic deletion of OST-PTP in mice results in hypoglycemia and protection from obesity and glucose intolerance [216].

## Therapeutic approaches to DKD

Despite significant advances in glycemic and blood pressure management, DKD often remains a progressive and largely irreversible condition. Management involves a combination of pharmacological approaches, renal replacement therapy for advanced disease, and lifestyle modifications, such as dietary interventions. Standard therapies include inhibition of RAAS using angiotensin converting enzyme (ACE) inhibitors or angiotensin receptor blockers (ARBs), which remain foundational for reducing intraglomerular pressure, proteinuria, and cardiovascular risk. Combination strategies have been explored, including dual ACE inhibitor and ARB therapy and the addition of aldosterone antagonists to ACE inhibitors or ARBs. The former showed no added benefit with increased risks of hyperkalemia, hypotension, and renal failure, whereas the latter can increase hyperkalemia risk and therefore demands rigorous monitoring [217–219]. Recent advances have established sodium-glucose cotransporter 2 (SGLT2) inhibitors as a cornerstone of DKD therapy due to their dual benefits. These drugs substantially slow the decline in kidney function and reduce major cardiovascular outcomes, independent of glycemic control [220, 221]. Indeed, landmark clinical trials including DAPA-CKD, EMPA-KIDNEY, and CREDENCE have validated their renoprotective effects and safety profile in patients with CKD; DAPA-CKD and EMPA-KIDNEY included patients with and without diabetes, whereas CREDENCE enrolled patients with T2DM, spanning moderate to advanced stages [221–223].

Additional agents such as glucagon-like peptide-1 (GLP-1) receptor agonists [224, 225], the nonsteroidal mineralocorticoid receptor antagonist finerenone [226], and emerging treatments further complement this armamentarium. GLP-1 receptor agonists have established renal and cardiovascular protection as demonstrated by the FLOW trial, in which semaglutide significantly reduced major kidney disease events, cardiovascular events, and all-cause mortality in patients with T2DM and CKD [227]. Meta-analyses and dedicated kidney outcomes trials establish that these agents reduce albuminuria, slow eGFR decline, and substantially lower the risk of major adverse cardiovascular events and cardiovascular death in patients with DKD [228, 229]. Finerenone exhibits anti-inflammatory and antifibrotic effects, and results from the FIDELIO-DKD and FIGARO-DKD trials position finerenone as a key add-on to maximize renoprotection and cardiovascular benefit, particularly in patients with persistent albuminuria despite optimal RAAS inhibition [230–232]. Emerging treatments being explored include antifibrotic agents (pirfenidone, pentoxifylline) [233, 234], JAK/STAT inhibitors (baricitinib) [235], and mitochondrial/oxidative stress modulators (NAD+ donors, bardoxolone methyl) [236, 237], which are at various stages of clinical testing and may inform future precision-based approaches. Targeted drug delivery to the kidney has also gained attention as a strategy to enhance efficacy and reduce systemic toxicity. Nanoparticle-, prodrug-, antibody-, and ligand-based carriers are being developed to concentrate therapeutics within specific renal compartments such as the glomerulus and proximal tubule [238]. By reducing systemic exposure, these systems aim to improve therapeutic indices; however, challenges remain due to the size and charge selectivity within the glomerulus and limited in vivo validation.

Moreover, recent advances in artificial intelligence, including in machine learning and deep learning (AI/ML/DL), are transforming drug discovery. Beyond accelerating target identification, molecular design, and virtual screening, AI-driven approaches enable the integration of high-dimensional structural, biochemical, and functional datasets to interrogate complex signaling networks underlying DKD [239]. AI/ML/DL workflows have already been applied to PTP1B and SHP2 inhibitor discovery, providing evidence that AI-guided strategies may help overcome historical barriers to targeting phosphatases, including highly conserved catalytic pockets across the PTP family [240–242]. By facilitating the identification of selective binding modes, allosteric sites, and optimized physicochemical properties, these approaches support a renewed view of classical PTPs as tractable therapeutic targets.

Collectively, these pharmacologic and delivery-based strategies aim to delay renal decline and reduce albuminuria, but reversal of established kidney injury is rare once it begins [243, 244]. This difficulty reflects the multifactorial nature of DKD, which is governed by intersecting metabolic, hemodynamic, and inflammatory pathways. To address this complexity, combination therapies are increasingly viewed as a more comprehensive strategy than single-agent intervention [245, 246]. Rather than relying on monotherapy, combination approaches employ multiple pharmacological agents that engage distinct molecular pathways, potentially offering additive or synergistic benefits [247, 248]. Encouragingly, emerging evidence suggests that DKD remission (a return to the physiological rate of eGFR decline with minimal or resolved albuminuria) may be achievable with directed combination therapy in select settings [247]. Consistent with this, a growing number of clinical trials are evaluating combination approaches for DKD, with some of the most recent efforts testing SGLT2 inhibitors together with aldosterone synthase inhibitors [249, 250].

### PTPs as therapeutic targets

Building on this paradigm, recent efforts have turned toward previously untapped molecular targets. Among these, PTPs have emerged as promising candidates due to their regulatory roles in pathways central to DKD pathogenesis, such as insulin signaling, oxidative stress, and inflammation. Advances in small-molecule development have begun to overcome historical challenges in PTP inhibitor specificity, opening the door to novel therapeutic strategies that may complement or enhance current standards of care. Earlier drug discovery efforts focused on selective PTP-active-site inhibitors, but these approaches were largely unsuccessful due to the high conservation of catalytic sites among PTPs and the positively charged nature of the active site [251]. For many years, PTPs were deemed “undruggable”, and to date, no selective PTP inhibitors are approved for clinical use, despite multiple candidates progressing through preclinical and early clinical development (Table 3). More recently, drug discovery efforts have expanded beyond active-site-only approaches to encompass strategies that exploit PTP allosteric sites, bivalent ligands that target both the active site and adjacent pockets, and targeted protein degradation. Small-molecule inhibitors with specificity for individual PTPs have shown encouraging results in preclinical models, and several candidates are currently under clinical investigation primarily in oncology, suggesting that PTP modulation could complement existing therapies and more effectively address the complex pathophysiology of DKD (Table 3).

**Table 3:** Pharmacological modulators of the classical PTPs

PTP target	Modulator name/code	Mode of action	Disease context	Clinical trial phase	References
*PTPRB*	VE-PTP-IN-1	Active site	N/A	Discovery	[252]
	Razuprotafib (AKB-9778)	Active site (competitive)	Diabetic retinopathy, retinal vein occlusion, ocular hypertension, glaucoma	2	[253–255]
*PTPRD*	7-BIA	Active site	Neuropathic pain, addiction	Preclinical	[256, 257]
*PTPRE*	cyt-PTPε Inhibitor-1 (Compound 62)	Active site	Bone formation	Preclinical	[258]
	Alendronate*	Active site oxidation	Osteoporosis	4	[259–261]
*PTPRG*	NAZ2329	Allosteric, dual	Cancer	Preclinical	[262]
*PTPRJ*	18E1 mAb	Agonistic	Diabetic nephropathy, renal injury	Preclinical	[81, 263]
*PTPRS*	DJ001	Allosteric	Stem cell regeneration	Preclinical	[264]
	Intracellular Sigma Peptide	Competitive	Multiple sclerosis	Preclinical	[265]
*PTPRZ*	NAZ2329	Allosteric, dual	Cancer	Preclinical	[262]
	MY10	Unknown	Alcohol intake	Preclinical	[266]
*PTPN1*	JTT-551	Mixed-type	Obesity	Preclinical	[267]
	IONIS-PTP-1BRx	Antisense-based oligonucleotide	T2DM	1	[268]
	ABBV-CLS-484	Active site, dual	Cancer	1	[269]
	KQ-791	Active site	T2DM	1	[270]
	Trodusquemine (MSI-1436)	Allosteric	Obesity, T2DM, cancer	1	[271, 272]
	Ertiprotafib (PTP112)	Protein aggregation	T2DM	2	[273]
	ISIS 113715	Antisense-based oligonucleotide	T2DM	2	[274, 275]
*PTPN2*	TP1L	PROTAC-mediated degradation	Cancer	Preclinical	[276]
	ABBV-CLS-484	Active site, dual	Cancer	1	[269]
*PTPN4*	Alendronate*	Active site oxidation	Osteoporosis	4	[259–261]
*PTPN6*	SHP1 activator 1 (Compound 3n)	Allosteric activator	N/A	Discovery	[277]
	TPI-1	Active site	Cancer	Preclinical	[278]
	M029	Covalent Allosteric	Cancer	Preclinical	[279]
	Sodium stibogluconate (Pentostam)	Active site	Cancer	1	[280]
*PTPN7*	MurA-IN-1	Active site	Cancer	Preclinical	[281]
*PTPN11*	DML189	Tyrosine-reactive covalent	N/A	Discovery	[282]
	SHP2-D26	PROTAC-mediated degradation	Cancer	Preclinical	[283]
	NSC-87877	Active site	Cancer	Preclinical	[284]
	PHPS1	Active site	DKD, AKI, cardiovascular disease	Preclinical	[24, 285, 286]
	GDC-1971	Allosteric	Cancer	1	[287]
	PF-07284892 (ARRY-558)	Allosteric	Cancer	1	[288]
	TNO155	Allosteric	Cancer	2	[289]
	JAB-3312	Allosteric	Cancer	3	[290]
*PTPN13*	LTV-1	Active site	Autoimmune diseases	Preclinical	[291]
*PTPN22*	PTPN22-IN-1	Allosteric	Cancer	Preclinical	[292]
	NC1	Allosteric	Autoimmune diseases	Preclinical	[293]
	LYP-IN-1	Active Site	Autoimmune diseases	Preclinical	[294]

For each PTP, inhibitors are listed according to their most advanced stage in the drug discovery pipeline, progressing from discovery to preclinical development and clinical trial phases (phases 1–4). PTPs not listed currently have no known inhibitors

*N/A*: not applicable or not yet reported in a specific disease context

*Alendronate was reported to inhibit *PTPRE* (PTPε) and *PTPN4* (PTP-MEG1) in vitro, but it was not clinically developed as a PTP inhibitor [295, 296]

Among the classical PTPs with a defined role in DKD, we highlight five for which pharmacological modulation is currently feasible: *PTPN11 (*SHP2), *PTPN1 *(PTP1B), *PTPN6 *(SHP1), *PTPRB *(VEPTP), and *PTPRJ *(CD148). These phosphatases are both mechanistically implicated in DKD and pharmacologically tractable. For most, this tractability takes the form of catalytic inhibition, and several inhibitors have advanced to clinical trials (Table 3). CD148 is a notable exception, for which the protective phenotype favors an activation strategy rather than inhibition; we include it to illustrate that pharmacological modulation of PTPs need not be restricted to enzyme blockade.

*PTPN11* (SHP2) inhibitors have recently shown substantial promise. Small-molecule SHP2 inhibitors have been pursued since the mid-2000s, when early screening campaigns identified some of the first selective inhibitors [284], laying the groundwork for the subsequent development of allosteric and active-site compounds. Since then, several candidates have been evaluated in phase I, II, and III clinical trials, primarily in solid tumors [287–290]. Although oncology-focused trials dominate the current landscape, emerging preclinical data suggest broader therapeutic relevance in kidney disease. In STZ-induced type 1 and *db/db* type 2 diabetic mouse models, the small molecule allosteric SHP2 inhibitor SHP099 markedly reduces albuminuria and BUN, indicating renoprotective effects beyond oncology applications [56]. Separately, the active-site inhibitor PHPS1 has shown protective effects across multiple injury models. In *db/db* diabetic mice, PHPS1 decreases albuminuria and alleviates fibrosis and renal inflammation [24], and it is similarly protective in models of acute kidney injury [285] and atherosclerosis [286]. Moreover, Novartis initiated a phase I clinical trial to assess the pharmacokinetics of the allosteric SHP2 inhibitor TNO155, a structural analog to SHP099, in patients with mild to severe renal impairment; however, the study was later withdrawn [297]. More recently, novel modalities have emerged for targeting SHP2, including covalent inactivation via electrophilic tyrosine modification [282] and PROTAC-mediated protein degradation [283], which may offer improved selectivity or durability over conventional inhibitors. Collectively, these findings highlight SHP2 as a promising target for DKD, though further clinical investigation in metabolic disease contexts is warranted.

Like SHP2, *PTPN1* (PTP1B) is a well-studied therapeutic target for diabetes and its complications due to its established role as a negative regulator of insulin and leptin signaling in vivo [298]. Over the 2000s-2010s, several phase I and II clinical trials evaluated PTP1B inhibitors, including the small molecule ertiprotafib and the aminosterol trodusquemine (MSI-1436) for T2DM and obesity [271, 273–275]. However, none advanced to regulatory approval due to challenges including poor oral bioavailability, insufficient efficacy, and safety limitations. Additionally, early efforts were hindered by the high sequence similarity between the catalytic domains of PTP1B and its close homolog TCPTP, raising concerns about achieving selectivity [299]. Alternative approaches to small molecules, such as antisense oligonucleotides (ASOs) targeting PTPN1 mRNA, exhibit high target specificity. Indeed, in obese, insulin-resistant rhesus monkeys, PTP1B ASO treatment improved insulin sensitivity [300], and in overweight or obese patients with T2DM, a second-generation ASO (IONIS-PTP-1BRx) lowered HbA1c, improved glycemic measures, and reduced body weight [268]. These findings reinforce the viability of PTP1B as a therapeutic target despite challenges in developing small-molecule inhibitors. Recently, a novel dual PTP1B/TCPTP inhibitor has entered phase I clinical trials for patients with advanced or metastatic solid tumors [269, 301]. While dual inhibition may broaden target engagement, its utility in DKD treatment remains unclear. PTP1B inhibition improves renal outcomes in preclinical models, but evidence for TCPTP inhibition in DKD is not established. This gap highlights an opportunity to investigate whether dual targeting of these phosphatases would confer therapeutic advantages in kidney injury.

*PTPN6* (SHP1) regulates the insulin signaling pathway and plays a well-characterized role in modulating podocyte architecture and function [53, 302]. Several categories of SHP1 inhibitors have been reported, including allosteric modulators and covalent cysteine-targeting compounds. However, a principal barrier to inhibitor development is poor selectivity over the closely related phosphatase SHP2, among other PTPs. Sodium stibogluconate (Pentostam) is a longstanding treatment for Leishmaniasis; however, it was never FDA-approved [280, 303]. It was reported to preferentially inhibit SHP1 with inhibitory activity against SHP2 and PTP1B, exhibiting differential sensitivities across these phosphatases [304]. Despite interest in its use in the context of cancer, its clinical use has declined over time because of significant toxicity and the widespread emergence of antimonial resistance in *Leishmania* [305]. In oncology, SHP1 has been pursued in both directions: inhibition to relieve immune cell suppression and activation to restore its tumor-suppressor function [277, 278]. Recent progress has led to the development of the novel allosteric SHP1 inhibitor M029, which shows selective inhibition in vitro and efficacy in preclinical, oncology-focused studies [279]. In contrast, in the diabetic kidney the therapeutic rationale is inhibition, since SHP1 contributes to podocyte injury and impairs insulin signaling. Given its mechanistic involvement in both metabolic and cytoskeletal regulation within the glomerulus, SHP1 represents a compelling target, and continued preclinical investigation is warranted to evaluate its therapeutic potential in DKD.

*PTPRB* (VEPTP) is highly expressed in renal endothelial cells and inducible genetic deletion improves several histopathological features associated with DKD, including glomerular matrix accumulation, glomerulosclerosis, and podocyte and slit diaphragm integrity [50]. Although inhibition of VEPTP using the antibody mAb 109.1 did not improve albuminuria or BUN levels in vivo, it did improve renal expression profiles of markers of endothelial activation, fibrosis, inflammation, and injury [67]. Beyond the kidney, VEPTP inhibition has been clinically evaluated using the small-molecule competitive inhibitor AKB-9778, which advanced to phase II trials for diabetic retinopathy and other ocular diseases [253–255]. Notably, in a preliminary report, patients with diabetes treated with AKB-9778 demonstrated improvements in select ocular endpoints and a modest reduction in urinary albumin-to-creatinine ratio (uACR) among participants with albuminuria [306]. Together, these findings suggest that while VEPTP inhibition alone may not provide complete renoprotection, it holds promise for targeting specific endothelial-driven mechanisms of DKD. Thus, VEPTP inhibition may serve as an adjunctive therapy when combined with agents targeting complementary pathways, a strategy supported by continued discovery-stage efforts to develop next-generation VEPTP inhibitors [252].

*PTPRJ* (CD148) is expressed in the renal vasculature, including within the glomerulus [307], where it is an established negative regulator of growth factor signaling, notably EGFR and VEGFR2, modulating angiogenic responses and endothelial function [263, 308]. Since CD148 acts protectively in this setting, the therapeutic strategy is target activation rather than inhibition. This approach is supported by two preclinical studies using the agonistic anti-CD148 antibody 18E mAb. In a mouse model of renal injury by uninephrectomy with chronic angiotensin II infusion, 18E mAb treatment lowered plasma creatinine and albuminuria and mitigated pathohistological changes, including reduced glomerulosclerosis and preserved podocyte number [263]. In a diabetes-specific setting, 18E mAb treatment in an STZ-induced diabetic mouse model likewise improved albuminuria and ameliorated mesangial matrix expansion [81]. Although no clinical trials currently evaluate CD148-targeted agents, its capacity to modulate EGFR and VEGFR2 signaling suggests that pharmacological CD148 activation may constitute a complementary strategy in DKD.

Taken together, these studies underscore the therapeutic promise of targeting specific PTPs in DKD. While most candidates remain in preclinical or oncology-focused clinical stages of development, the mechanistic rationale for their use in renal disease is compelling. Given that DKD is a multifactorial, progressive disorder involving dysregulation across several signaling pathways, approaches that combine compatible pharmacological therapies to restore balance within these disrupted networks may offer added or synergistic benefit. Further investigation into their cell-type-specific roles, context-dependent effects, and potential for combination therapy is warranted to realize their translational potential in DKD.

## Conclusions

DKD remains the leading cause of end-stage renal failure and an area of urgent unmet medical need. While existing therapies mitigate disease progression, they rarely reverse renal injury, underscoring the importance of identifying upstream modulators of DKD pathophysiology. Classical PTPs are integral modulators of cellular signaling pathways implicated in DKD, such as insulin signaling, inflammation, oxidative stress, and cytoskeletal remodeling. This review outlines the diverse roles of PTPs in DKD, highlighting both well-characterized and emerging candidates. Advances in pharmacological targeting, including the development of allosteric inhibitors with improved selectivity, have reignited interest in PTPs as druggable targets, with representative compounds summarized in Table 3. Although most candidates remain at preclinical or oncology-focused clinical stages, accumulating evidence suggests that selective PTP modulation may offer a novel and complementary approach to existing DKD therapies. Future research must bridge preclinical findings with translation, focusing on defining the mechanisms driving disease and exploring combinatorial strategies to unlock the therapeutic promise of PTP-targeted therapies in DKD.

## Data Availability

No datasets were generated or analysed during the current study.

## Abbreviations

ACE: Angiotensin converting enzyme
AGE–RAGE: Advanced glycation end products–receptor for advanced glycation end products
AI: Artificial intelligence
AKI: Acute kidney injury
Akt: Akt serine/threonine kinase
AMPK: Adenosine monophosphate-activated protein kinase
Ang -1: Angiopoietin-1
ARB: Angiotensin receptor blocker
BUN: Blood urea nitrogen
CKD: Chronic kidney disease
DEG: Differentially expressed gene
DKD: Diabetic kidney disease
DL: Deep learning
eGFR: Estimated glomerular filtration rate
EGFR: Epidermal growth factor receptor
eNOS: Endothelial nitric oxide synthase
ER: Endoplasmic reticulum
FAK: Focal adhesion kinase
FOXO3: Forkhead box O3
GBM: Glomerular basement membrane
GFR: Glomerular filtration rate
GH: Growth hormone
GLP-1: Glucagon-like peptide-1
GWAS: Genome wide association studies
HOMA-IR: Homeostatic model assessment of insulin resistance
IGF-1: Insulin-like growth factor-1
IL-34: Interleukin-34
ILK: Integrin-linked kinase
IR: Insulin receptor
IRS1/2: Insulin receptor substrates 1 and 2
JAK/STAT: Janus kinase/signal transducer and activator of transcription
KIBRA: Kidney and brain protein
LATS1/2: Large tumor suppressor kinases 1 and 2
MAPK: Mitogen-activated protein kinase
ML: Machine learning
mTOR: Mechanistic target of rapamycin
NADPH: Nicotinamide adenine dinucleotide phosphate
NFκB: Nuclear factor kappa B
NO: Nitric oxide
PEPCK: Phosphoenolpyruvate carboxy kinase
PI3K: Phosphatidylinositol-3 kinase
PIPKIγ: Phosphatidylinositol-4-phosphate 5-kinase type 1 gamma
PDGFRβ: Platelet-derived growth factor receptor beta
PTECs: Proximal tubular epithelial cells
PTKs: Protein tyrosine kinases
PTPs: Protein tyrosine phosphatases
RAAS: Renin-angiotensin-aldosterone system
ROS: Reactive oxygen species
SGLT2: Sodium-glucose cotransporter 2
SNP: Single nucleotide polymorphisms
SRC: Proto-oncogene tyrosine-protein kinase Src
T1DM: Type 1 diabetes
T2DM: Type 2 diabetes
TGF-β: Transforming growth factor beta
TIE2: TEK receptor tyrosine kinase
TRIP6: Thyroid receptor-interacting protein 6
uACR: Urinary albumin to creatinine ratio
VEGF: Vascular endothelial growth factor
VEGFR2: Vascular endothelial growth factor receptor 2
WNT3a: Wingless-type MMTV integration site family, member 3A
YAP: Yes-associated protein
ZO-1: Zonula occludens-1
